# The Influence of Contextual Factors on the Relative Age Effect in Male International Rugby Union: The Impact of Sociocultural Influences and Playing Position

**DOI:** 10.3390/children9121941

**Published:** 2022-12-10

**Authors:** Paolo Riccardo Brustio, Adam Leigh Kelly, Corrado Lupo, Alexandru Nicolae Ungureanu

**Affiliations:** 1 Department of Clinical and Biological Sciences, University of Turin, 10126 Turin, Italy; 2 NeuroMuscularFunction, Research Group, School of Exercise & Sport Sciences, University of Turin, 10126 Turin, Italy; 3 Centre for Life and Sport Sciences (CLaSS), Faculty of Health, Education, and Life Sciences, Birmingham City University, Birmingham B15 3TN, West Midlands, UK; 4 Department of Medical Science, University of Turin, 10126 Turin, Italy

**Keywords:** RAE, rugby football union, talent development, athlete development, cultural context, talent identification

## Abstract

The purpose of this study is not only to establish whether the relative age effect (RAE) exists in male international rugby union players, but also to investigate the impact of sociocultural influences (i.e., northern and southern hemispheres) and playing position (i.e., backs, forwards, and scrum-halves). The birth date and the playing position of 7144 senior male professional rugby players included in the rosters of the season 2020–2021 were collected from the top 10 nations of the World Rugby rankings (i.e., Argentina, Australia, England, France, Ireland, Japan, New Zealand, Scotland, South Africa, and Wales). Data were analyzed using a chi-square goodness-of-fit test to compare the observed and expected birth quarter (Q) distributions. Results showed that relatively older players were overrepresented in all the sample (*p* < 0.001; Q1 = 28.8% vs. Q4 = 20.3%). In players competing in both hemispheres, the RAE was weak despite a more pronounced RAE emerging for southern players. In addition, the RAE was present in backs and forwards, but inconsistent for scrum-halves. In general, the data suggest that relatively older players may be more likely to reach expertise at senior levels than their later-born peers, and that the effect was consistent in different sociocultural contexts as well as in backs and forwards.

## 1. Introduction

Talent identification and development programs are current topics throughout the majority of sports governing bodies/federations. These organizations aim to create pathways from the initial enrolment into sports at ‘grassroots’ level (i.e., entry) to the adult professional level (i.e., expertise) [1]. However, the decision-making process to identify and develop talented athletes with the prerequisites and potentialities to become future high-level athletes is complex, not straightforward, and highly challenging [1,2]. Indeed, a complex interplay between the performer (e.g., physiological factors; psychosocial characteristics; technical and tactical skills), environment (e.g., impact of parents, coaches, and peers; sociocultural influences; organizational structures), and task (e.g., participation history; opportunities and access to facilities and resources; playing position) influence the pathway towards senior success and long-term athletic development (for a review see [1,2,3]).

One selection bias that can arise during these processes is the relative age effect (RAE). The RAE reflects the (dis)advantages and outcomes resulting from an interaction between the selected dates and birthdate [4,5]. During childhood, young athletes are banded according to (bi)annual-age groups to facilitate equitable learning opportunities and competitive experiences by limiting intragroup physical and cognitive differences [6]. However, an interval of one or two years within the same age cohort can create developmental differences and participation and attainment among peers, which benefits relatively older athletes while disadvantaging relatively younger athletes. One of the underlying causes of the RAE has been explained through physiological biases (i.e., the maturation-selection hypothesis) [4,7]. Accordingly, players born in the early months near the selection date are likely at a physical advantage due to normative growth and/or physical characteristics [7]. Relatively older athletes also possess more playing experiences in the early stages of participation, which can aid their short-term performance [8]. Furthermore, the social agents model introduced by Hancock and colleagues [9] explains the RAE as a social phenomenon. The model outlines that different social agents, including parents, coaches, and athletes themselves may positively or negatively impact the RAE (i.e., the selection of some players at the expense of others). For example, coaches can impact relatively older athletes’ self-esteem by giving them, for instance, more attention during practice or more playing time. Indeed, when relatively older athletes receive positive feedback regarding their performances, the same are more likely to have high self-perception and self-expectations and, thus, motivated to continue participating in sports activities [10]. These aspects, related to the self-fulfilling prophecy, may lead athletes to perform at levels consistent with expectations. In addition, Kelly and colleagues [11] used the personal assets framework to explain the immediate, short-, and long-term developmental outcomes of the RAE, which highlights the need to better understand how relative age must be examined across different timescales and sociocultural contexts to better understand the aforementioned mechanisms. Overall, despite the true mechanisms of the RAE remaining inconclusive, what is known is that it can limit the possibility of selecting relatively younger talents with long-term potential, which has significant implications on performance, participation, and personal development [11].

It is plausible to suggest that rugby union’s contact and invasive nature combined with the high physical demand required during competition may exacerbate the RAE [12]. A player being chronologically older than peers may lead to performance requirement advantages [13], including rucking, running with the ball, scrummaging, and tackling. Consequently, coaches and practitioners may be more prone (consciously or unconsciously) to select relatively older athletes due to their greater physical performance capacities at the youth level [13].

In this regard, several national studies in the rugby union context, especially considering European countries [12,14,15,16,17,18,19,20,21], who identified an over- and under-representation of the relatively older and younger players, respectively, highlighted the influence of contextual factors such as gender, age group, competition level, sociocultural factors, and playing position. In male rugby, a birthdate inequality was observed in UK rugby league during initial enrolment at grassroots level that starts from the Under-7 stage until the senior age group [21]. Similarly, in Welsh rugby, the RAE was presented from the Under-7 stage to Under-19, where the percentage of players born in the first three months from the selection date (i.e., Q1 = 29%) was higher than the percentage of players born in the last three months from the selection date (i.e., Q4 = 21%) [19]. Additionally, the RAE increased when selection steps and performance levels increased, indicating that when fewer places on the squad occurred, the RAE increased [21]. Indeed, the odds ratios (ORs) identified a significant risk of the RAE increasing between players born in Q1 and Q4 when the performance levels increased (e.g., in Under-16 categories: district OR = 2.64; regional OR = 4.67; national OR = 11.96) [19].

Interestingly, when the RAE was explored during the transition from academy to the professional level in rugby, a possible reversal effect of relative age [22] occurred [23,24]. Specifically, while the proportion of relatively older players was higher at the academy level (i.e., Q1 = 41.5% vs. Q4 = 8.47%), the proportion of relatively younger players who reached success at professional levels was higher compared to the relatively older players (i.e., Q1 = 20% vs. Q4 = 50%) [23]. This finding was confirmed by Kelly and colleagues [18], where relatively younger players were about four times more likely to achieve professional or international status during their senior career once they entered the talent pathway. This phenomenon, commonly explained by the underdog hypothesis [22,25], may lead the relatively younger peers to have greater potentiality for later success in comparison with their relatively older peers [26].

Despite the RAE seeming conclusive at the youth level, findings remain mixed at the senior level depending on sociocultural context. For example, in the UK, no significant difference was highlighted in the quartile distributions within senior cohorts (e.g., Q1 and Q4; ~25%) [18]. On the contrary, rugby union players born near the selection date in Italy were about 1.5 times more likely to reach the first and second elite division even if the index decreases as age increases [20]. Contrastingly, from a French perspective, the RAE had a weak or no magnitude effect [14,16]. Although, another study on the French senior league showed that the RAE existed for forwards (especially for back row forwards) but not for backs [16]. When analyzing the top 10 internationally ranked teams over 20 years, Jones and colleagues [27] revealed the traditional skewed distribution for backs (favoring Q1) and reversal RAE for forwards (favoring Q4). In addition, during a cross-cultural comparison, the RAE was observed in Australian, English, New Zealand, and South African professional players [15], whilst South Africa was the only country with a pronounced RAE according to all playing position (i.e., forwards and backs). Moreover, differences in the playing philosophy (i.e., technical, and tactical model of performance) were reported to exist between northern and southern hemispheres (e.g., more offloads, more tries in southern hemisphere), as well as in the strength and conditioning practice (e.g., emphasis on strength and power training or on objectively determining training loads) [28,29]. Together, these findings suggested that the possible differences in national culture and playing position are important considerations to examine while exploring who is at risk of the RAE [12].

To date, only one study, to the authors’ knowledge, investigated the RAE by adopting a cross-cultural approach and analyzing senior male professional rugby, including Australian, English, New Zealand, and South African male players [15]. Therefore, investigating this issue may be important to better understand rugby players’ birth distribution, and the consequent national federation policy associated to the talent identification system. As a consequence, and in consideration of the possible differences in the national cultural context and playing position, this study aimed to: (a) evaluate the potential differences between the countries of the northern and southern hemispheres, and (b) examine possible differences between playing positions based on backs, forwards, and scrum-halves. Due to the heterogeneity in RAE results at the senior level in rugby union, no a priori hypothesis was formulated. Nevertheless, we expected to find possible differences due to the divergent technical and tactical model of performance between northern and southern hemispheres. Additionally, we expected to find RAE magnitude difference when considering players’ position according to Kearney [15].

## 2. Materials and Methods

Data were downloaded from the open web https://www.ultimaterugby.com/ on 1 December 2021. The database contains information about male teams competing in the most prominent nations in World Rugby (i.e., according to the most recent Rugby World Cup results in 2019 and the press coverage of domestic competitions). To explore the RAE at the highest levels of competition, for the current study, we arbitrarily focused our analysis on the top 10 nations included into the World Rugby rankings (https://www.world.rugby/tournaments/rankings/mru accessed on 1 December 2021). Thus, only data about senior male professional rugby players competing in the first- and second-division teams of Argentina, Australia, England, France, Ireland, Japan, New Zealand, Scotland, South Africa, and Wales were included. Thus, the birth date and the playing positions of 7144 senior male professional rugby players included in the rosters of the season 2020–2021 were collected with the approval of the local institutional review board. Data are available from the web (public domain), thus no permission was needed.

## 3. Statistical Analysis

Consistent with the selection year from each participating country (i.e., January to December: Argentina, Australia, France, Ireland, New Zealand, and South Africa; September to August: England, Scotland, and Wales; April to March: Japan), players’ birth dates were categorized into four quarters (i.e., Q1, Q2, Q3, and Q4) and semesters (S1 and S2). Moreover, the time of birth (TB) was calculated to identify how far a player was born from the selection date using the following formula: TB = (birth week−0.5)/52. For more details on this method, please see the works from Brustio and colleagues [6,20,30].

Data were analyzed by merging all the players and grouping them according to countries of the northern (i.e., England, France, Ireland, Japan, Scotland, and Wales) and southern (i.e., Argentina, Australia, New Zealand, and South Africa) hemispheres to test the impact of sociocultural influences. Moreover, players were categorized into their playing position based on backs, forwards, and scrum-halves to examine the influence of playing position.

Differences between the observed (i.e., our data) and expected (i.e., 25% for each quartile) [15] quartile distributions were assessed using chi-square goodness-of-fit tests (χ^2^). An expected birth distribution of 25% for each quartile was chosen considering the databases containing the birthdates of different nationality athletes. Cramer’s V was calculated to determine the effect of magnitudes. The threshold values for effect size statistics were: 0.06 ≤ V for a trivial effect; 0.06 < V ≤ 0.17 for a small effect; 0.17 < V < 0.29 for a medium effect; and V ≥ 0.29 for a large effect. Comparisons between the first and last quartile (Q1 vs. Q4) and between the first and second semester (S1 vs. S2) were calculated using odds ratios (ORs) and 95% confidence intervals (CIs). Moreover, to investigate the RAE phenomenon further, Poisson regression for analyzing low count data was used to consider birth week distribution as a continuous variable. The relative odds (i.e., index of discrimination—ID) of being selected for a player born in the first week versus the last week of the competition year were calculated [6,30]. All data were analyzed with a custom script written in MATLAB R2020b (MATLAB, R2020b, MathWorks: Natick, MA, USA, 2022). Results were considered statistically significant when *p* < 0.05.

## 4. Results

Table 1 reports the birth quartile distribution, the chi-square (χ^2^) statistics, and the ORs for all players competing in the top 10 national professional rugby union leagues and considering the northern and southern hemispheres and playing position (i.e., all playing positions together as well as backs, forwards, and scrum-halves separately).

**Figure 1:**
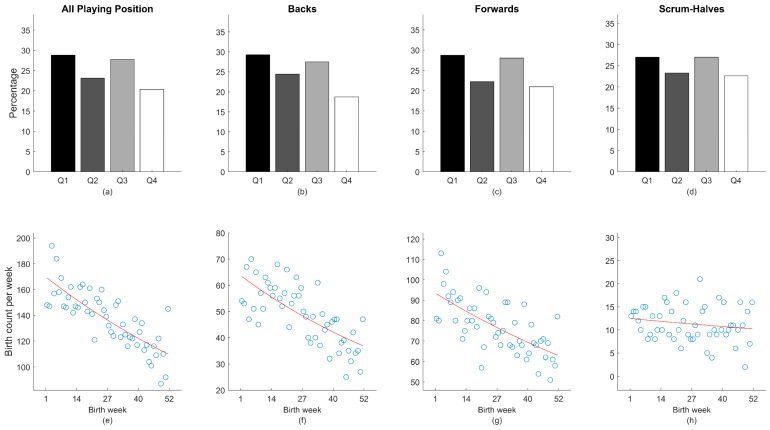
Birth quartile percentage distributions (**a**–**d**) and a scatterplot of birthdate frequency by week (**e**–**h**) presented individually for all playing positions, backs, forwards, and scrum-halves. The red line represents the best fit of the Poisson regression.

When considering all players without distinction of hemisphere and playing position, a birth skewed distribution was observed (χ^2^ = 136.044, *p* < 0.001) with a small effect size in the overall samples (V = 0.08; see Figure 1a). The ORs showed an increased likelihood of relatively older players being selected in Q1 compared to the Q4 (OR = 1.42, CI [1.29, 1.56]). Poisson regressions confirmed these results (y = e^(5.14−0.44x)^, R^2^ = 0.64, *p* < 0.001), whereby the ID showed that, overall, players born in the first week after the selection year were 1.56 times more likely to be included in the senior rosters than those born in the last week of the selection year (Figure 1e). When considering players’ positions, there was a significant difference between quartile distribution with a small effect size (V ranged = 0.08–0.09) in backs (χ^2^ = 65.524, *p* < 0.001; Figure 1b) and forwards (χ^2^ = 75.259, *p* < 0.001; Figure 1c) but not in scrum-halves (χ^2^ = 3.973, *p* = 0.264; Figure 1d). The ORs showed an increased likelihood for relatively older players being selected (i.e., players born in Q1) in backs (1.56, CI [1.33, 1.83]) and forwards (1.37, CI [1.21, 1.55]). The Poisson regressions confirm these results for all playing positions, which included: (a) backs (y = e^(4.17−0.58x)^, R^2^ = 0.53, *p* < 0.001), (b) forwards (y = e^(4.54−0.40x)^, R^2^ = 0.46, *p* < 0.001), and (c) scrum-halves (y = e^(2.52−0.20x)^, R^2^ = 0.03, *p* = 0.17). The ID highlighted that backs and forwards born in the first week after the selection date was 1.78 (Figure 1f) and 1.49 (Figure 1g) times more likely to be included in the rosters than those born in the last week of the selection date, respectively. See Figure 1 for a visual inspection of the overall players’ data, considering birth quartile and birth week distribution.

Small effect sizes (V ranged = 0.09–0.11) were apparent in the overall sample when comparing the northern and southern hemispheres (see Table 1). In the northern hemisphere, ORs showed that players born in Q1 were 1.32 times more likely to be selected (CI [1.18, 1.47]). Contrastingly, ORs were higher in players competing in the southern hemisphere, where the likelihood of relatively older players being selected in Q1 compared to the Q4 was 1.67 (95% CI [1.41, 1.98]). When comparing the northern and southern hemispheres, data suggested a more pronounced RAE for backs and forwards competing in the southern hemisphere (V ranged = 0.10–0.14) than in the northern hemisphere (V ranged = 0.09–0.11). Accordingly, significant ORs revealed that backs and forwards of the southern hemisphere born in Q1s were approximately 1.7 times more likely to be selected than those born in Q4s (*p* < 0.001). In the northern hemisphere, Q1s were about 1.5 times more likely to be selected than Q4s (*p* < 0.001). A similar percentage trend was observed in Q1s and Q4s when focused on the scrum-halves. The Poisson regressions also showed significant results for backs (northern hemisphere: y = e^(3.75−0.50x)^, R^2^ = 0.33, *p* < 0.001; southern hemisphere: y = e^(3.10−0.75x)^, R^2^ = 0.29, *p* < 0.001) and forwards (northern hemisphere: y = e^(4.09−0.28x)^, R^2^ = 0.23, *p* < 0.001; southern hemisphere: y = e^(3.51−0.64x)^, R^2^ = 0.31, *p* < 0.001) but not for scrum-halves (northern hemisphere: y = e^(2.08−0.07x)^, R^2^ < 0.01, *p* < 0.67; southern hemisphere: y = e^(1.49−0.29x)^, R^2^ = 0.02, *p* = 0.28). The IDs highlighted that northern hemisphere backs and forwards born in the first week after the selection date were 1.65 and 1.33 times more likely to be included in the rosters than those born in the last week of the selection date, respectively. In the southern hemisphere, the ID highlighted that backs and forwards born in the first week after the selection date were 2.12 and 1.89 times more likely to be included in the rosters than those born at the last week of the selection date, respectively.

## 5. Discussion

The present study aimed to explore the quartile distributions in the teams from the top 10 nations, according to the World Rugby rankings, considering the sociocultural influences (i.e., the northern and southern hemispheres) and playing positions (i.e., backs, forwards, and scrum-halves). The key findings of the study were that: (a) in the teams of top 10 World Rugby rankings, data revealed a skewed birth date distribution (favoring relatively older players), (b) the comparison between northern and southern hemisphere data suggests a weak RAE, both in northern and southern hemisphere, with a more pronounced RAE in the southern hemisphere, and (c) independent of the sociocultural context, the RAE was more prevalent for backs than forwards and inconsistent for scrum-halves.

When considering all teams in the top 10 World Rugby rankings, data suggested a persistent but weak RAE (V = 0.08). Findings revealed a skewed birthdate distribution favoring relatively older players (i.e., approximately 29% and 20% in Q1 and Q4, respectively). Players of Q1 were 1.42 times more likely to achieve professional status at the senior level than those of Q4. Nevertheless, it is necessary to highlight that compared to studies on young national pathways, we found a lower effect size, suggesting a weaker effect at the senior level. Indeed, previous studies at the senior international level have found contrasting results. More specifically, in rugby, current research has showed a persistent RAE [18,24], no RAE [20], and a reversal effect of the RAE [28]. For example, a skewed birthdate distribution favoring relatively older players was found in French [18] and Italian [20] players at the senior level. In comparison, however, there was no significant difference in the quartile distributions within both English senior premiership and international players [22]. Even if we found a weak magnitude of the RAE in this senior context, it can be assumed that, according to the selection and maturation hypothesis [10], the selection process at the senior level that is in favor of relatively older players may be explained, in part, by the critical role of physical characteristics important for achieving successful performances. This may be particularly true considering the nature of rugby union. During competitions, high physical demand is required due to contact and its invasive nature. Consequently, being chronologically older and, thus, probably more physically mature than peers may confer performance advantages [13]. Moreover, based on the theoretical model provided by Hankook et al. [9], different social agents, including parents, coaches, or the athletes themselves may have exacerbated RAE. The implications of the RAE in younger age groups are undoubtedly perpetuated through perceptions of athlete competence, including athletes’ perceptions of themselves (i.e., Galatea effect), their coaches (i.e., Pygmalion effect), and their parents (i.e., Matthew effect) [9]. Initially, parents may influence the RAE by encouraging more frequently the relatively older athletes to take up sports (i.e., Matthew effect). Meanwhile, coaches might place greater expectations (e.g., more attention during the training sessions) on relatively older athletes and consequently advantage them (i.e., Pygmalion effect). Finally, older athletes, due to the higher expectation of parents and coaches, may increase their self-efficacy (e.g., perceive themselves as being more gifted) and be more motivated to work harder to meet expectations [30]. Overall, data suggest and confirm that the RAE at the senior international level is symptomatic of selection problems observed at the youth level [20], and thus the RAE mechanisms must be better understood to develop relevant solutions.

When focused on possible sociocultural differences (i.e., comparison between the northern and southern hemispheres), findings suggested that the countries in southern hemisphere (i.e., Argentina, Australia, New Zealand, and South Africa) showed a higher RAE than those from the northern hemisphere (i.e., England, France, Ireland, Japan, Scotland, and Wales). Independent of the players’ playing position, the proportion of relatively older players in this study was 1.62 (southern hemisphere) and 1.32 (northern hemisphere) times higher than relatively younger players; however, it is important to note that both hemispheres’ RAE showed low magnitude, as well as no difference in effect size. The southern hemisphere represents a test bench for new rules (World Rugby experimental ruleset) and a cutting-edge technical and tactical performance [31]. Thus, it can be speculated that the highest level of international rugby performance requires the greatest level of technical, tactical, physical, and anthropometric skills to be achieved as soon as possible, which likely favors relatively older players with a physical and technical, and tactical advantage. However, this possible explanation is only speculated and needs investigation in further studies. Moreover, despite the various cut-off dates across different countries (i.e., January to December, September to August, April to March), the RAE remained consistent. Indeed, similar findings have shown how the RAE remains prevalent in youth rugby union irrelevant of the change of cut-off date [12]. Therefore, practitioners and policymakers should be cautious of independent cut-off dates and how they can influence player development opportunities at both youth and senior levels.

Findings related to players’ position indicated that, independent from the sociocultural context, the RAE appeared in backs and forwards and was inconsistent for scrum-halves. Interestingly, contrasting results are found in the literature when the effect of players’ position was evaluated in relation to the RAE. For instance, Jones and colleagues [27] observed differences in the birth distribution concerning players’ position among the world’s best rugby union players, whereby a skewed birthdate distribution favored relatively older players for backs whereas there was a reversal birthdate distribution for forwards (i.e., favor younger players). In contrast, when analyzing Australian, English, New Zealand, and South African professional players at the senior level, Kearney [15] indicated that the RAE existed for forwards but not backs. In this present study, we found that at the senior level, selections of both backs and forwards were affected by the RAE, but not for scrum-halves. Due to the peculiarities of scrum-halves (e.g., the different game demands in terms of running intensity [32] and collision magnitude [33]) as well as the anthropometric profile differences (e.g., leaner and shorter players) when compared to backs and forwards, both at senior [34] and junior level [35,36], it is possible to suggest that coaches and stakeholders may be more poised to select backs and forwards to benefit from a greater body mass and strength rather than scrum-halves, which may further explain the how physiological characteristics make athletes more vulnerable to the RAE.

## 6. Limitations

It is important to acknowledge that the present study is affected by some limitations. Firstly, only one competition year (i.e., season 2021–2022) was examined for only top 10 nations. Indeed, results from a longitudinal perspective may have provided more concrete findings, whilst observing more diverse countries could have highlighted more impact on the sociocultural influences (e.g., the impact of sport popularity). Secondly, our dataset only included male international rosters. The difference in the RAE across female cohorts observed in other national contexts highlights the need to investigate gender differences at the international level of rugby union. However, it is also important to highlight that the development of female rugby union talent pathways and senior competition is developing rapidly. Thus, one should learn from some of the male RAE lessons when designing and implementing new organizational structures to create more appropriate settings. Finally, it is necessary to consider that we only investigated the RAE based on sociocultural influences and playing position, and thus did not consider other developmental factors linked with players selection. For example, studying the RAE alongside other individual constraints (e.g., performance match statistics, physical performances, maturation status) may further inform the potential mechanisms in a broader view.

## 7. Conclusions

The present results add a broader international overview to the RAE in rugby union literature. Overall, our data suggested that relatively older players may be significantly more likely to be selected in the senior rosters than their later-born peers. This effect was consistent in different sociocultural contexts (despite being more pronounced for the southern hemisphere) as well as for playing positions (i.e., more pronounced for backs). According to these findings, decision making during the selection process should favor a long-term vision in both the northern and southern hemispheres. Moreover, selection criteria should consider the athletes’ long-term potential rather than their current performance capabilities. As a consequence, this approach could positively reform the RAE by widening the potential talent pool and preventing the risk of hindering relatively younger athletes from reaching their maximum potential.

## Figures and Tables

**Table 1:** Birth quartile distribution, chi-square value, and odds ratio analysis considering the different playing positions.

	Population	N	Q1	Q2	Q3	Q4	χ^2^	*p*	V	V Category	OR Q1 vs. Q4	OR S1 vs. S2
All playing position	All sample	7144	28.8	23.1	27.8	20.3	136.044	<0.001	0.08	Small	1.42 [1.29, 1.56]	1.08 [1.01, 1.15]
	Northern Hemisphere	4859	28.1	20.8	29.7	21.4	122.551	<0.001	0.09	Small	1.32 [1.18, 1.47]	0.96 [0.88, 1.04]
	Southern Hemisphere	2285	30.2	28.1	23.6	18.1	79.560	<0.001	0.11	Small	1.67 [1.41, 1.98]	1.40 [1.25, 1.57]
Backs	All sample	2545	29.3	24.4	27.5	18.7	65.524	<0.001	0.09	Small	1.56 [1.33, 1.83]	1.16 [1.04, 1.30]
	Northern Hemisphere	1737	29.7	20.6	30.1	19.6	66.574	<0.001	0.11	Small	1.51 [1.25, 1.83]	1.01 [0.89, 1.16]
	Southern Hemisphere	808	28.5	32.7	22.0.	16.8	47.327	<0.001	0.14	Small	1.69 [1.27, 2.26]	1.57 [1.29, 1.92]
Forwards	All sample	4011	28.7	22.3	28.0	21.0	75.259	<0.001	0.08	Small	1.37 [1.21, 1.55]	1.04 [0.95, 1.14]
	Northern Hemisphere	2719	27.6	20.6	29.8	22.0	63.622	<0.001	0.09	Small	1.25 [1.08, 1.46]	0.93 [0.83, 1.03]
	Southern Hemisphere	1292	31.2	25.9	24.2	18.7	40.811	<0.001	0.10	Small	1.67 [1.33, 2.08]	1.33 [1.14, 1.55]
Scrum-Halves	All sample	588	27.0	23.3	27.0	22.6	3.973	0.264	0.05	-	1.20 [0.86, 1.65]	1.01 [0.81, 1.27]
	Northern Hemisphere	403	25.1	23.1	27.5	24.3	1.713	0.634	0.04	-	1.03 [0.70, 1.52]	0.93 [0.70, 1.22]
	Southern Hemisphere	185	31.4	23.8	25.9	18.9	5.935	0.115	0.10	-	1.66 [0.92, 2.98]	1.23 [0.82, 1.85]

Notes: Q1, first quartile percentage; Q2, second quartile percentage; Q3, third quartile percentage; Q4, fourth quartile percentage; χ^2^, chi-square value; V, Cramer’s V effect size; OR, odds ratio and 95% confidence intervals [95% CI]; Q1 vs. Q4, first versus the last quartile; S1 vs. S2, first versus the last semester.

## Data Availability

The data presented in this study are available on the data file S1.

## Supplementary Materials

The following supporting information can be downloaded at: https://www.mdpi.com/article/10.3390/children9121941/s1.
